# Sumateran wild boar (*Sus scrofa vittatus*) meat antibody production as immunodiagnostic reagent candidate

**DOI:** 10.14202/vetworld.2019.477-482

**Published:** 2019-04-01

**Authors:** Melani Wahyu Adiningsih, Retno Damajanti Soejoedono, Rahmat Setya Adji, Dwi Desmiyeni Putri, Trioso Purnawarman, Hadri Latif, Okti Nadia Poetri

**Affiliations:** 1 Study Program of Veterinary Public Health, IPB Graduate School, Bogor Agricultural University, Indonesia; 2 Indonesia Agricultural Quarantine Agency, Indonesia; 3 Department of Animal Diseases and Veterinary Public Health, Faculty of Veterinary Medicine, Bogor Agricultural University, Indonesia; 4 Center of Veterinary Research, Bogor, Indonesia; 5 Department of Animal Husbandry, Faculty of Animal Husbandry, State Polytechnic of Lampung, Indonesia

**Keywords:** antibody, enzyme-linked immunosorbent assay, rabbit, reagent, Sumateran wild boar

## Abstract

**Aim:**

Meat authentication gives significance values in view of religious, food safety, public health, quality assurance, and legal concern. Most of the meat authentication is based on molecular assay; a simpler method to authenticate meat is needed to develop. An immunoassays technique may offer a solution for simpler test. The aim of our current study was to develop a polyclonal antibody of *Sus scrofa vittatus* (Sumateran wild boar) as an immunodiagnostic reagent candidate.

**Materials and Methods:**

Three male New Zealand white rabbits were used in this study for antibody production. Antigen used was meat extract of Sumateran wild boar, each rabbit was immunized with meat extract antigen (0.5 mg/ml) emulsified in Freund’s complete adjuvant at a 1:1 (v/v) ratio as much as 1 ml at subcutaneous route. Booster was carried out 3 times with interval time of 14 days, using meat extract antigen emulsified in Freund’s incomplete adjuvant at a 1:1 (v/v) ratio. Serum samples were taken every week, start from 1 week after the first immunization up to 1 week after the third booster. Antibody purification was performed using ammonium sulfate precipitation and Protein A. The presence of specific antibody was determined using agar gel precipitation test and enzyme-linked immunosorbent assay, while purified specific IgG was characterized using sodium dodecyl sulfate-polyacrylamide gel electrophoresis method.

**Results:**

Specific antibody was detected at 14 days after the first immunization and still detected until 2 weeks after the third booster. Highest absorbance of specific antibody was detected 1 week after the third booster.

**Conclusion:**

The present study demonstrated that specific antibody of Sumateran wild boar is favorable to be produced in rabbit and showed that antibody produced is applicable to detect Sumateran wild boar meat antigen in immunodiffusion assay, indicating that it is promising as a reagent candidate in immunodiagnostic assay/kit.

## Introduction

Consumption of meat and its product is increasing each year worldwide; however, adulteration of meat has been a long time issue. In China, “*hang a sheep head, sell vinegar*” is a widely known proverb. Meat adulteration affects food safety, quality, and many other aspects and has becoming a public focus [1]. To overcome this problem, various analytical methods based on physical, chemical, anatomical, histological, and biological approaches are being utilized to identify meat species [2]. *Sus scrofa vittatus* (Sumateran wild boars) are superabundant in Sumateran forest [3], this condition has led to exploitation for commercial purpose [4]. High number of Sumateran wild boars population increases wild boar hunting, resulting in an abundant availability of wild boar meat in the food market with extremely cheap price. The macroscopic similarity of wild boar meat and beef has prompted the local people to abuse this situation by selling wild boar meat in traditional market as beef. Antibodies are an important tool used by many investigators in their research and have led to many medical advances. Mammalian sera represent a remarkable and economical source of immunoglobulins widely used in diagnostic and therapeutic applications [5-7]. In biochemical and biological research, polyclonal antibodies are routinely used as ligands for the preparation of immunoaffinity columns [8] and as coating or labeling reagents for the qualitative and quantitative determination of molecules in a variety of assays such as enzyme-linked immunosorbent assay (ELISA), double diffusion, radial immunodiffusion, western blot, and radioimmunoassays [9-11].

Meat authentication gives significance value in view of religious, food safety, public health, quality assurance, and legal concern. Most of the meat authentication is based on molecular assay. A simpler method to authenticate meat is needed to develop an immunoassays technique, may offer a solution for simpler test. This research aimed to produce and characterize specific polyclonal antibody from Sumateran wild boar meat as an immunodiagnostic reagent candidate to develop rapid test tool.

## Materials and Methods

### Ethical approval

This research has been approved by the Animal Care and Use Committee of Research and Community Services Institution, Bogor Agricultural University, with approval number: 090/KEH/III/2018.

### Experimental animal

Experimental animals used in this study were three New Zealand white rabbits aged 10-16 weeks with an average body weight of 2.5 kg obtained from the Indonesian Animal Husbandry Research Institute, Ciawi, Bogor, Indonesia.

### Research design

This study was divided into six parts such as (a) preparation of antigen, (b) production of antibody, (c) antibody detection using agar gel precipitation test (AGPT), (d) antibody detection using ELISA, (e) purification of antibody, and (f) characterization of antibody.

### Preparation of antigen

Antigen used was meat extract of Sumateran wild boar, preparation antigens were carried out according to the method of Potter [12]. Five volumes of the wash solution (1% Triton X-100, 50 mM KC1, and 5 mM Tris, pH 8.0) was then added to the ground muscle and further homogenized with stomacher at high speed. The homogenate was then centrifuged at 10,960 g for 15 min, and the supernatant was discarded. The pellets were resuspended in an equal volume of the wash solution added directly to the centrifuge bottles. These were rehomogenized and then recentrifuged. This procedure was repeated 8-10 times, or until the residue turns almost white (slight yellow tinge). All these steps were carried out at 4°C.

The pellets were transferred to a 4 L plastic beaker. Three volumes of prechilled 95% ethanol were added to the pellet, which is then broken up with a gloved hand. The tissue was then collected over a Buchner funnel, diameter 26 cm, containing a Whatman No. 1 filter paper (24 cm), mounted on a 4 L filter flask to which suction was applied. The filtrate was discarded. The tissue was transferred to the beaker, and this procedure was repeated 3 times. Next, the same procedure was conducted as above except that diethyl ether was substituted for the ethanol. After the four washes, the powder was left to dry overnight on the filter paper, with suction. The dry powder was weighed and stored at 4°C.

The powder was extracted overnight with a 15:1 (v/w) ratio of 1 M KC1, 25 mM Tris, pH 8.0, 0.1 mM CaCl_2_, and 0.1 mM DTT. The extract was centrifuged at 10,960 g for 10 min at 4°C. This supernatant was set aside, and the pellet was then resuspended by adding 1 M KCl directly to the centrifuge bottles, in a ratio of 7.5:1 (v/w of starting tissue). The mixture was then centrifuged at 10,960 g for 10 min at 4°C. The pellet was discarded, and the supernatant from this centrifugation was pooled with the previous one. The pH of this solution was lowered to 4.6 with 1 N HCI. The precipitated was collected by centrifuging at 7020 g for 10 min at 4°C. This pellet can be stored at −20° to be used later. The pH of the supernatant from this centrifugation was adjusted to 8.0 with 1 N KOH, and 230 g of ammonium sulfate per liter (40% saturation) was slowly added with constant stirring, the pH being maintained between 7 and 8. This solution was then centrifuged at 10,960 g for 10 min at 4°C; the pellet was discarded. The supernatant was brought to 60% (NH_4_)_2_SO_4_, saturation by adding 125 g of ammonium sulfate per liter as described above. The supernatant from this spin was discarded, and 40-60% (NH_4_)_2_SO_4_ pellet was then suspended in a minimal volume of 10 mM imidazole, pH 7.0, 50 mM KC1, 0.1 mM CaCl_2_, 0.1 mM DTT, and 0.02% NaN_3_ and then dialyzed.

### Production of antibody

Antibody production was performed in three New Zealand white rabbits. Meat extract antigen (0.5 mg/ml) was emulsified at a 1:1 (v/v) ratio with Freund’s complete adjuvant and 1 ml was injected into the subcutaneous on day 1 for immunization initiation. Booster was carried out using emulsifying antigen suspension with a 1:1 (v/v) ratio with Freund’s incomplete adjuvants on days 14, 28, and 42 after the first immunization. Antibody presence was analyzed by AGPT and ELISA; serum samples were collected on days 21, 35, and 49 after the first immunization. Final serum samples collection was performed on 14 days after the third booster. Bleeding is performed by taking blood intracardially after the rabbits had been anesthetized with a ketamine (35 mg/kg BB) and xylazine (5 mg/kg BB) mixture. The collected blood was prepared as follows: The blood samples were stored at room temperature (±25°C) for an hour and continued storage at 4°C overnight. The obtained serum was separated manually by aspiration and was completed by centrifugation at 2500 rpm for 15 min. The obtained serum was stored in 1.5 ml polypropylene microcentrifuge tubes and stored at −20°C until use.

### Antibody detection using AGPT

AGPT was carried out on the serum samples using the methods described by Okwor *et al*. [13]. It was performed using immunodiffusion plates with 10 ml of 1% agarose (Sigma, USA CAS 9012366) containing 8% sodium azide at pH 7.2 ± 0.1. Using a template and cutter wells of 4 mm diameter and 4 mm interspace (apart) were cut, the plates were set up with groups of six wells in a circle surrounding a center well. The peripheral wells were filled with the serum samples to be tested, while the center well was filled with the antigen. The plates were incubated at 37°C and read at 24, 48, and 72 h under diffused light. Positive serum samples showed a line of precipitation between the serum and antigen well.

### Antibody detection using indirect ELISA

ELISA procedure was similar to conventional protocols by Kong *et al*. [14] with modification. Briefly, the coating antigen was dissolved in coating buffer (0.05 M, carbonate-bicarbonate, pH 9.6), and then, 100 μL was added to each well of the 96-well plates that were subsequently incubated at 4°C overnight. The wells were then washed 3 times with phosphate buffer saline tween 0.05%. After washing, 100 μL of serum (1:100 dilutions) were added to each well, and the plates were incubated at 37°C for 1 h. After washing 3 times, horseradish peroxidase-labeled goat anti-rabbit IgG (1:5000) as much as 100 μL was added to each well, and the plates were again incubated at 37°C for 1 h. After 3 times washing, 100 μL 3,3′,5,5′-tetramethylbenzidine substrate was added to the wells. The enzymatic reaction proceeded for 15 min at 37°C in darkness and then was stopped with addition of 50 μL sulfuric acid (2 M) per well. The results were read at 450 nm using a microplate reader.

### Purification of antibody

Antibody purification was performed in two steps using ammonium sulfate precipitation [15] followed by Protein A purification. Ammonium sulfate precipitation was done as follows: 4.1 M ammonium sulfate solution was added to serum at ratio 1:1 and incubated overnight at 4°C, and then, the mixture was centrifuged at 3.000× g for 30 min. Obtained pellet was reconstituted by phosphate-buffered saline (PBS) pH 7.4 to obtain initial amount; subsequently, dialysis was performed by preparing a precipitated serum in a dialysis tube (Spectra/Por, USA) and stirred in pH 7.4 of PBS for 24 h at 4°C and each 8 h PBS solution was replaced. The second step was followed using Protein A purification kit (Sigma, USA) according to the manufacturer’s instructions. Protein concentration was determined by spectrophotometer using coefficient 1.36 at 280 nm wavelength [16].

### Characterization of antibody

Purified antibody was analyzed by sodium dodecyl sulfate-polyacrylamide gel electrophoresis (SDS-PAGE) method using 12% polyacrylamide concentration for separating gel and 4% for stacking gel [17]. SDS-PAGE was carried out using horizontal electrophoresis method (Amersham ECL gel box, GE 17 Healthcare) using 12% precast acrylamide gel concentration (GE, Healthcare). Sample was dissolved in 2× sample buffer (2.5 ml 0.5M Tris pH 6.8; 2.0 ml glycerol; 4.0 ml SDS 10%; 0.2 ml 2-mercaptoethanol; 0.2 ml bromphenol blue 1.0%; and 3.2 ml aquadest), total volume 10.0 ml with a ratio of 1:1. Sample concentration is 15 μg. Sample suspension was heated for 5 min at 90-100ºC. To adapt the gel, the precast acrylamide gel was placed on an electrophoresis machine (ECL gel box); then, a running buffer was added (25 mM Tris; 192 mM glycine; 0.1% SDS, pH 8.3) as much as 190.0 ml, then electrophoresed at a voltage of 160 V for 12 min. The power supply is turned off, then the protein marker (Piercenet, USA) and the sample is inserted into each gel hole as much as 20.0 ul, then electrophoresed with a voltage of 160 V for 60 min. The gel is then removed from the cassette and stained with solution (40.0% methanol; 10.0% acetic glacial; and 0.1% blue commission); then, the stain is removed by destaining solution (40.0% methanol and 10.0% acetic glacial).

## Results and Discussion

### Production of antibody

**Figure-1:**
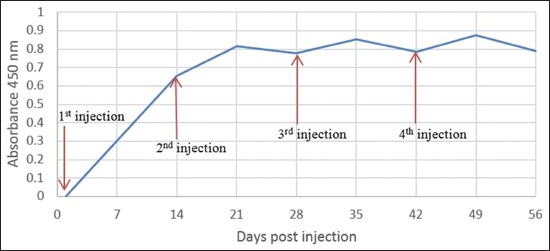
Absorbance value after antigen injection was determined by enzyme-linked immunosorbent assay.

The presence of a specific antibody in ELISA was indicated by absorbances values >0.5. Specific antibodies started to be detected in the 2^nd^ week post-first immunization (pi1); then, the absorbance tends to increase due to booster. Absorbance at day 14 pi1 was 0.665, increasing into 0.817 1 week after the first booster, then decline into 0.778 at 2 weeks after the first booster, and the trend is repeated. Our result indicated that antibody level tends to increase for 7 days post-booster, and then begin to decline and increases again after another booster. Highest absorbance of specific antibody was detected 1 week after the third booster (Figure-1).

**Figure-2:**
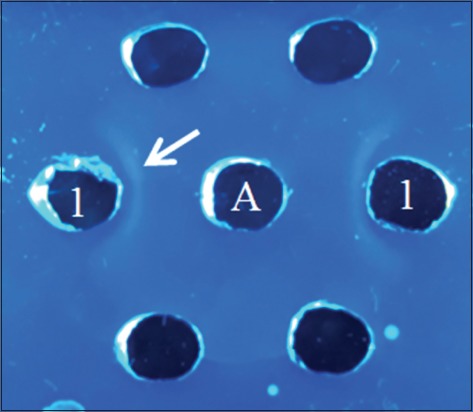
Antibody specificity test with agar gel precipitation test; 1 - Antigen *Sus scrofa vittatus*; B. Serum.

AGPT was conducted to confirm that the antibodies produced are able to bind with Sumateran wild boar meat antigen. The results are performed in Figure-2, whereas the precipitation line indicates that antibodies produced were homologous with Sumateran wild boar meat antigen.

### Purification and characterization of antibody

**Figure-3:**
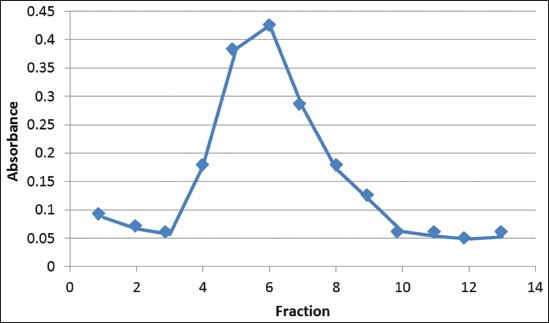
Pattern of antibody fraction after purification.

In the present study, the antibody purification was done in two steps using ammonium sulfate (4.1 M) and Protein A purification kit (Sigma). Based on the UV/Vis spectrophotometer, antibody purification using ammonium sulfate and Protein A produced several fractions, which higher antibody concentration is at fraction number 5, 6, and 7 (Figure-3). Purified antibody in two step purification resulted in concentration of 1.457 mg/ml (Table-1).

**Table-1:** Absorbance rate and antibody level after purification.

Fraction	Antibody	

	Absorbance value (280 nm)	Level of antibodies (mg/ml)
1	0.086	0.063
2	0.066	0.049
3	0.057	0.042
4	0.176	0.129
5	0.384	0.282
6	0.425	0.313
7	0.281	0.207
8	0.173	0.127
9	0.117	0.086
10	0.062	0.049
11	0.053	0.039
12	0.049	0.036
13	0.052	0.038
	Total	1.457

**Figure-4:**
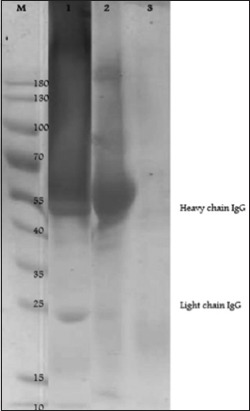
Profile of sodium dodecyl sulfate-polyacrylamide gel electrophoresis antibody before purification (1), after purification with ammonium sulfate (2), and after two steps purification with ammonium sulfate and Protein A purification kit (3). Protein marker (M).

Specific antibody of Sumateran wild boar meat was characterized using SDS-PAGE method by determining its molecular weight. In the current study, we characterized two kinds of purified antibody: (1) Antibody purified with ammonium sulfate precipitation and (2) antibody purified sulfate and Protein A precipitation. Electrophoresis results of SDS-PAGE showed that 1^st^ purified antibody has seven protein bands and 2^nd^ purified antibody has three protein bands (Figure-4). Molecular weight determination was conducted by making a linear regression curve on the calculation of relative mobility value (Rf) and protein molecular weight molecule logarithm. We get equation of y=−0.0651x+2.4886; R^2^=0.9905 (Table-2). Based on the equation, the molecular weight of standard whole IgG was 159.28 kDa, heavy chain IgG was 53.48 and 48.81 kDa, and light chain IgG was 22.55 kDa, respectively (Table-3).

**Table-2:** Determination of equations based on linear curves.

Rf (cm)	BM (kDa)	Log BM
4.58	180	2.26
5.32	130	2.11
6.91	100	2.00
9.26	70	1.85
11.59	55	1.74
13.59	40	1.60
15.03	35	1.54
17.07	25	1.40
20.72	15	1.18
22.04	10	1.00

Y= -0.0651x+2.4886

**Table-3:** Calculation of protein molecular weight band in SDS-PAGE analysis of antibody.

Purification methods	Rf (cm)	BM (kDa)	Log BM
Ammonium sulfate	4.4	159.28	2.20
	7.69	97.27	1.99
	10.84	60.66	1.78
	11.68	53.48	1.72
	12.29	48.81	1.69
	13.11	43.16	1.63
	17.44	22.55	1.35
Protein A	11.68	53.48	1.72
	12.29	48.81	1.69
	17.44	22.55	1.35

SDS-PAGE=Sodium dodecyl sulfate-polyacrylamide gel electrophoresis

In the present study, we produced a polyclonal antibody of Sumateran wild boar meat as an immunodiagnostic reagent. In general, monoclonal antibodies were the main choice for immunodiagnostic reagents [18]; however, polyclonal antibodies has several advantages such as high affinity, able to identify high homologous proteins of immunogen proteins, can be used to filter protein targets of non-immunogen species, able to detect denatured proteins, and able to detect more than one epitope so the detection is more sensitive. To produce an antibody of Sumateran wild boar meat, we immunized rabbits with initial immunization and 3 times booster. The first immunization induces primary immune which introduces antigen to B cells producing low-level antibody and memory B cells, whereas booster induces secondary immune response producing a higher level of antibody [19]. Specific antibody is detected at 14 days after the first immunization and still detected until 2 weeks after the third booster. The highest absorbance of specific antibody was detected 1 week after the third booster or 49 days.

Antibody level was determined by ELISA indicated by absorbance value/optical density value. Absorbance value at day 14 after the first immunization showed 0.655 was considered that the serum at a point of time has specific antibody of Sumateran wild boar meat, and the absorbance value tends to increase after the booster. Highest absorbance level is 0.879 reached at 7 days after the third booster; nonetheless, absorbance value of antibody after boosting in each sampling time tends not to be different. The absorbance value obtained indicating that the amount of antibodies produced was not high; however, it is enough to be able to detect Sumateran wild boar meat antigen on AGPT. Level of antibody produced depended on antigenicity of the antigen, and antigenicity is depended on genetic distance of the antigen and host, molecular weight, structure of molecule, epitope density, and its degradability [19].

According to Putri *et al*. [20], antibody production in laboratory animals has become an essential part of research projects. To produce antibodies, investigators are confronted with a lot of complex choices, some of which may be critical for success. To obtain high-titer, high-specificity antibody, and concerned in animal welfare were main goals in antibody production. Antibody produced should be induced by characterized antigen and also be characterized before used as a reagent in the immunodiagnostic test. Our study proves that a specific antibody of Sumateran wild boar meat can be produced in rabbit.

Before characterized, antibodies must be separated from other components in the serum. According to Murphy *et al*. [21], separation of antibodies from other components in the serum can be done by purification. In the present study, we purified antibody in one step using ammonium sulfate precipitation only and two-step purification using ammonium sulfate – Protein A precipitation. Purification results were characterized by SDS-PAGE to determine the molecular weight (Figure-4).

Molecular weight of IgG rabbit was 150 kDa, with two heavy chains (about 50 kDa each) and two light chains (about 25 kDa each) under non-reducing condition [22]. IgG has a molecular weight of monomer structure 146,000 Da, and it is the main antibody of the secondary immune response. Chemical treated IgG molecules, such as SDS, will break the disulfide bond and cause the IgG molecules to break down into four separate polypeptide chains. These chains are “heavy” chain which has a molecular weight of about 50 kDa and two other “light” chains which have a molecular weight of about 25 kDa. This is in line with the study by Sadeghil *et al*. [23] which showed that SDS-PAGE analysis results a band contained protein with approximately 50 kDa MW that was rabbit IgG heavy chains. The light chain of rabbit IgG appeared as a diffused band of 20-30 kDa molecular weights. It was likely that diffused band of the light chain could be related to the different level of deglycosylation of protein during manipulation process.

Our result showed that purified antibody using ammonium sulfate precipitation only, resulting seven protein bands, and it is likely some substances such transferrin, albumin, and other proteins at molecular weight 97.27 kDa, 60.66 kDa, and 43.16 kDa. According to Johnson [22], transferrin has a molecular weight of 75 kDa, and albumin has a molecular weight of 60 kDa. While purified antibody using ammonium sulfate – Protein A precipitation resulting three protein bands, and it is likely that band at 55 kDa is a heavy chain and band at 25 kDa is a light chain. Our result indicating that more step in purification will result in a more purified antibody.

Polyclonal antibody is an antibody which induced by injection whole or partial antigen. Polyclonal antibodies have a complex mixture of antibodies with different specificity, affinity, and isotype. Polyclonal antibodies have the function for binding various epitopes on the surface of the inducing antigen molecule [20]. AGPT result showed the appearance of a precipitation line near the well-containing antibody which is mean weakly positive, or it is likely that the antibody titer is low [24]. Despite low antibody titer, our results demonstrated that antibody produced was able to detect Sumateran wild boar meat antigen. To develop antibody as a reagent for an immunodiagnostic kit, it is likely that we need antibody at high titer; however, producing antibody in high titer was not our aim. Nonetheless, our study indicates that specific polyclonal antibody of Sumateran wild boar meat was promising as a reagent candidate to develop rapid test tools and favorable to be produced in rabbit.

## Conclusion

This present study demonstrated that specific antibody of Sumateran wild boar is favorable to be produced in rabbit. Specific antibody was first detected at 14 days after the first immunization and still detected until 2 weeks after the third booster. Our result also showed that antibody produced is applicable to detect Sumateran wild boar meat antigen in immunodiffusion assay, indicating that it is promising as reagent candidate in immunodiagnostic assay/kit.

## Authors’ Contributions

MWA carried out the research work; RDS and RSA planned, designed, and supervised the experiments; ONP and DDP supervised in the collection and of sample and laboratory work; TP and HL supervised in drafting and revising the manuscript critically for important intellectual content. All authors read and approved the final manuscript.
